# Decline in an Atlantic Puffin Population: Evaluation of Magnitude and Mechanisms

**DOI:** 10.1371/journal.pone.0131527

**Published:** 2015-07-15

**Authors:** Will T. S. Miles, Roddy Mavor, Nick J. Riddiford, Paul V. Harvey, Roger Riddington, Deryk N. Shaw, David Parnaby, Jane M. Reid

**Affiliations:** 1 Institute of Biological and Environmental Sciences, University of Aberdeen, Aberdeen, Aberdeenshire, United Kingdom; 2 Joint Nature Conservation Committee, Aberdeen, Aberdeenshire, United Kingdom; 3 Fair Isle Bird Observatory, Fair Isle, Shetland, United Kingdom; 4 Shetland Biological Records Centre, Shetland Amenity Trust, Garthspool, Lerwick, Shetland, United Kingdom; 5 Spindrift, Eastshore, Virkie, Shetland, United Kingdom; Norwegian Polar Institute, NORWAY

## Abstract

Determining which demographic and ecological parameters contribute to variation in population growth rate is crucial to understanding the dynamics of declining populations. This study aimed to evaluate the magnitude and mechanisms of an apparent major decline in an Atlantic Puffin *Fratercula arctica* population. This was achieved using a 27-year dataset to estimate changes in population size and in two key demographic rates: adult survival and breeding success. Estimated demographic variation was then related to two ecological factors hypothesised to be key drivers of demographic change, namely the abundance of the main predator at the study site, the Great Skua *Stercorarius skua*, and Atlantic Puffin chick food supply, over the same 27-year period. Using a population model, we assessed whether estimated variation in adult survival and reproductive success was sufficient to explain the population change observed. Estimates of Atlantic Puffin population size decreased considerably during the study period, approximately halving, whereas Great Skua population estimates increased, approximately trebling. Estimated adult Atlantic Puffin survival remained high across all years and did not vary with Great Skua abundance; however, Atlantic Puffin breeding success and quantities of fish prey brought ashore by adults both decreased substantially through the period. A population model combining best possible demographic parameter estimates predicted rapid population growth, at odds with the long-term decrease observed. To simulate the observed decrease, population models had to incorporate low immature survival, high immature emigration, or increasingly high adult non-breeding rates. We concluded that reduced recruitment of immatures into the breeding population was the most likely cause of population decrease. This study showed that increase in the size of a predator population does not always impact on the survival of adult prey and that reduced recruitment can be a crucial determinant of seabird population size but can easily go undetected.

## Introduction

Populations increase and decrease over time according to changes in demographic parameters such as survival and reproductive success [1–3]. Determining which demographic parameters are primarily responsible for variation in population growth rate is crucial to understanding population dynamics and underlying mechanisms [4–6]. Different demographic parameters do not all affect population growth rate equally and this variation has been examined in numerous populations of plants, reptiles, birds and mammals [7–12]. Demographic parameters are influenced by a variety of ecological factors, for example resource availability, predator abundance and climatic conditions [13–16]. Quantifying multiple demographic parameters and identifying the ecological factors that drive demographic variation are two valuable steps towards understanding population dynamics [2, 5, 6, 17–19]. Such a holistic approach, however, is difficult in studies of wild systems because it requires long-term datasets on population size, multiple demographic parameters and multiple potential ecological factors, which are often not feasible to collect.

Diagnosis of reasons for long-term decreases in population size is particularly valuable for ecological indicator species and small or declining populations that may receive conservation attention and management [20–23]. Seabirds and marine mammals are the top predators in marine systems and are consequently useful indicators of the state of inshore and offshore communities; seabirds also provide a tractable opportunity to study marine dynamics because they can be studied on land when they come ashore to breed [20, 24, 25]. In the UK, seabird populations have been monitored since 1986 through a nationally coordinated Seabird Monitoring Program (SMP). In the northeast Atlantic waters around Scotland, population sizes of most seabird species have decreased since 1986 [26, 27]. This environment receives considerable attention from fisheries, renewable and non-renewable energy development, and wildlife interest groups [28–30]. Understanding changes in populations of marine organisms, particularly those in long-term decline, is now highly relevant [31–32].

The survival of adult and young seabirds is greatly influenced by diet, during both the breeding and non-breeding seasons [33–37]. The reproductive performance and survival of many species breeding in the North Atlantic have been found to vary in close association with Lesser Sandeel *Ammodytes marinus* stock characteristics [26, 38–41]. In the northwest North Sea, in some years there appears to be no alternative high-energy prey and if Lesser Sandeels are not easily available then seabird breeding is either poor or fails completely [42,43]. Additionally, seabirds are vulnerable to predation by a range of mammalian and avian predators [26, 44, 45]. Island seabird assemblages have been considerably reduced by predation by introduced cats, rats and mice; also, many skua and gull populations heavily predate seabirds at their breeding colonies [26, 45, 46–48]. Very heavy predation of adults and chicks of several species by Great Skuas *Stercorarius skua* has recently been reported in northern Scotland [49–56]. Eradication of mammalian and avian predators from seabird islands has been used worldwide as an often effective conservation management strategy; however, prior to such action holistic evaluation of causes of population change is essential.

Many studies have demonstrated, implicitly or explicitly, the importance of trophic linking and top-down control mechanisms (e.g. predation) on seabird demographic rates, and how critical it is to consider such processes when assessing potential driving factors of population change, because effects can be substantial [14, 35, 40, 57–63]. Rarely, however, have effects of top-down control mechanisms, underlying demographic rates and bottom-up ecological drivers been evaluated simultaneously [64, 65]. One reason for this is that multi-year seabird data series describing multiple demographic and ecological variables from the same site are relatively rare. Measuring key variables for seabirds, especially those breeding at remote locations, is often impeded by logistical and financial limitations.

Natural and anthropogenic changes in ecological factors, such as predator abundance and food availability, can have important direct effects on demography but also indirectly affect seabird behaviour. This can reduce the likelihood of individuals being seen on land and hence impede measurement of demography, potentially leading to spurious inferences regarding demographic change [41, 66–68]. Change in seabird behaviour and detectability has not been widely studied, but is a critical consideration when demographic parameters are estimated by direct observation.

The aim of this study was to evaluate evidence of an apparent major decline in an Atlantic Puffin *Fratercula arctica* (hereafter Puffin) population in the light of variation in demography, resighting probability, predator abundance, and prey characteristics. We achieved this aim using a 27-year dataset from a multi-faceted SMP study to estimate changes in population size and in two key demographic rates: adult survival and breeding success. We then related the estimated demographic variation to two ecological factors hypothesised to be key drivers of demographic change; namely the abundance of the main predator of Puffins at the site (the Great Skua *Stercorarius skua*) and Puffin chick food supply, over the same 27-year period. In addition, we examined the potential causes and consequences on demographic estimates of a change in Puffin behaviour during this period. Using a population model, we assessed whether estimated variation in adult Puffin survival and reproductive rates were sufficient to explain the observed population change. We thereby evaluate the evidence for, and mechanisms causing, a major decline in a seabird population.

## Materials and Methods

This study was carried out with appropriate licensing and permissions for bird handling and ringing in the UK, issued by the British Trust for Ornithology.

### Study species and site

The Puffin is a medium sized auk Alcidae (350–600g) that breeds in the North Atlantic from France and the Gulf of Maine in the south to as far north as there is ice-free land, and that winters over vast areas of the North Atlantic and in the western Mediterranean [41]. Fair Isle (59°54′N, 01°62′W), Shetland, holds a population of Puffins that was most recently estimated to number approximately 11,000 breeding individuals (see Results), which nest in grassy cliffs around the island’s 30km of coastline. The main predator of Puffins on Fair Isle is the Great Skua, a large seabird (1150–1650g) that feeds primarily on fish, bird, mammal, and shellfish meat gained by direct predation, kleptoparasitism and scavenging [46]. Remains of Puffins killed by Great Skuas, instances of predation and of hunting of Puffins by aerial pursuit and ambush on foot by Great Skuas have been directly observed extensively during 1986–2013 (>>50 incidental observations annually; Fair Isle Bird Observatory staff pers. comm.) [53]. During this period, remains of Puffins killed by other predators on the island, including domestic and feral cats *Felis catus* and Great Black-backed Gulls *Larus marinus*, have been found relatively rarely (less than annually) and no direct evidence of predation of Puffins by any species other than Great Skuas has been observed.

### Population size

Total Puffin population size was estimated using a standardised protocol of synchronized island-wide counts of all adults visible on land, in the air and on the sea within 200m of the shore, between 16:00 and 20:00 on calm, dry days in the pre-laying period (late April) in 1986, 1989, 1995, 2000, 2001, 2007, 2009 and 2012 [69, 70]. These timings were selected because visible activity of breeding Puffins peaks in the evening and in late April most breeders have arrived at their breeding colonies but egg-laying has not yet commenced [41]. This timing can be assumed to exclude non-breeders, which mostly come ashore later, in June and July [41, 69, 71]. Daily checks for the first Puffins ashore on Fair Isle were made every year from late March onwards. The first mass-arrival of Puffins on land always occurred in mid-April and counts were made as soon as possible thereafter as weather permitted. The timing of first arrival and of the first pre-laying breeding activity of breeding Puffins on land did not advance or get later during the study period, so population counts were always made in late April and this timing was not adjusted. Island-wide counts were carried out simultaneously by a team of 6–20 observers covering pre-defined separate sections of Fair Isle’s coastline, thereby ensuring complete coverage of the entire coastline and Puffin colony. Counts were carried out multiple times (minimum = 3, as weather permitted) within each census year on calm evenings when Puffins were ashore. The highest recorded total within each year was used as the estimate of total Puffin population size, because this count minimises the proportion of individuals that were missed during a single count, for example because they were in burrows or further out at sea. This methodology follows a standard protocol established as part of the SMP [69]. It provides a rough estimate of total population size (measured as individual birds) rather than exact size, allowing large changes in population size to be detected but not small year-to-year discrepancies, which require data of higher resolution to detect. Methods of directly censusing numbers of active nest burrows of Puffins, such as quadrat or transect sampling [69] are not possible on Fair Isle, where the grassy cliffs suitable for Puffin breeding are mostly too steep to allow human access, thus maximum April count is the best feasible method.

### Predator population size and diet composition

The total number of breeding pairs of Great Skuas on Fair Isle was estimated annually from 1987 to 2013, by complete counts of apparently occupied territories (AOT = counting unit) across the whole island. An AOT was counted and marked (to avoid double counting) upon finding a nest scrape, eggs, chicks and/or an aggressively territorial pair of adults swooping on or striking an observer [46]. Annual AOT counts were made by systematic coverage of all areas of suitable habitat, repeated three times (minimum) in each breeding season [69].

The diet of breeding adult Great Skuas was assessed by identifying prey remains in regurgitated pellets of indigestible material, collected from all AOTs where a nest was found in 2011 and 2012. For each territory, a circular area of approximately 15m radius from the nest scrape was searched for pellets. Great Skua pairs defend their territories against conspecifics highly aggressively, thus pellets within territories can be confidently assigned to breeding pairs rather than non-breeding or transient individuals [46, 72]. Territories were visited every 15–20 days between May (egg laying) and mid-August (fledging), all pellets were collected and removed to prevent recounting, and all prey remains identified to the lowest possible taxon using established criteria [72–74].

Skua pellets are typically of broadly similar size, shape and structure irrespective of their content so pellet-finding is unlikely to have been biased towards particular prey types [75]. Pellet production varies according to prey-type, so the relative proportions of different prey-types in the diet were calculated using standardised values of total meals consumed of different prey-types (1 meal = quantity of food present in a bird’s proventriculus on its return from feeding) [49], rather than the (non-standardised) total pellet frequencies. Values of meals consumed were calculated by applying prey-specific correction factors to pellet frequencies, determined from studies of captive Great Skuas fed meals of different prey [72, 73]. Total numbers of meals consumed and proportional representation in the diet were calculated from total numbers of pellets collected in all territories for the following prey-type categories, encompassing the prey species that were encountered: European Storm-petrel *Hydrobates pelagicus*, Northern Fulmar *Fulmarus glacialis*, European Shag *Phalacrocorax aristotelis*, Black-legged Kittiwake *Rissa tridactyla*, Great Skua chicks, Common Guillemot *Uria aalge*, Razorbill *Alca torda*, Puffin, auks (including Common Guillemot, Razorbill, Black Guillemot *Cepphus grylle* and Puffin but species unidentifiable), seabird eggs (all species), unidentified / other bird (including passerine, unidentified gull/tern and unidentified bird sp.), fish (all species), mammal (Rabbits *Oryctolagus cuniculus*, Sheep *Ovis aries* carrion and Field Mice *Apodemus sylvaticus*) and other (including goose barnacle *Lepas* sp., squid Teuthida sp. and cuttlefish Sepiida sp.).

### Adult survival

To estimate apparent annual survival probabilities of adult Puffins, a sample of breeding adults caught at a focal sub-colony at Roskillie, Fair Isle, was marked using unique combinations of 3 colour-rings and one metal ring, each year in June and July during 1986 to 2013 (total of 477 individuals marked, annual mean of 17.0 ± 21.9 SD ranging from 0 in 1999, 2001 and 2003 to 111 in 1987, S1 Table). Individuals were caught by mist net or burrow net and classified as breeding adults only if they had more than 2 bill grooves (indicates age of 4 years or older) and were carrying fish when caught. Only such confirmed adults were colour-ringed, meaning that the sample included individuals of unknown exact age but that were 4 years or older (Puffins are not likely to breed until at least 4 years old and most do not breed until aged 6 years or older) [41]. Individuals caught that could not be classified as breeding adults were ringed with a uniquely coded metal ring and released, but were not colour-ringed and did not contribute to the survival study. Colour-ring resighting watches were carried out at the focal sub-colony every year from 1987 to 2013, usually in dry and calm conditions, between 1 April and 31 August, with an average effort intensity of three watches of approximately 2 hours per week throughout June and July and ad hoc observations at other times. A total of 1786 resightings of colour-ringed individuals was documented during the study period (sum of the total number of colour-ringed individuals resighted in each year. Annual mean of 64 individuals resighted, ranging from 11 in 2006 to 119 in 1990, S1 Table).

Up to the late 1990s, Puffins gathered in large numbers on the grassy cliff top of the colony meaning that colour-ring combinations could be read easily. However, thereafter Puffins increasingly gathered on the sea below the cliffs, impeding colour-ring resightings. When Puffins were ashore, they were extremely tolerant of the presence of humans at or near the colony, sometimes approaching to a distance of <1m. By 2005, large gatherings on land were rare and typically only formed close to dusk. Adult Puffins returning to the colony landed at their burrow entrance and ran quickly underground, and subsequently flew directly back to sea. From 2005 onwards, Great Skuas were commonly observed patrolling the cliff edge hunting Puffins and other seabirds. In an attempt to encourage Puffins to gather on land to allow ring resightings, before and during ringing and resighting sessions all Great Skuas standing near the Puffin colony were flushed (skua clearance) and the cliff top thereby temporarily cleared of predators for the entire duration of each session. This practice was first implemented on an ad hoc basis from 2007 to 2010 then systematically implemented during all resighting sessions from 2011 to 2013.

Capture-mark-recapture (CMR) models were fitted a priori to estimate apparent annual survival (φ) and resighting (recapture) probabilities (p) and hence to quantify temporal variation in φ and p during 1987 to 2013. Individual colour-ringed Puffins were recorded as present (observed) or absent (not observed) within each annual resighting period, with multiple resightings of an individual in any year equating to single encounters. When the rare loss of one or more rings meant that an individual’s identity was uncertain (approximately 10 individuals towards the end of the study) the full encounter histories of all possible individuals were excluded from analyses.

A fully year-dependent Cormack-Jolly-Seber (CJS) model was initially fitted [76] using MARK version 6.1 [77, 78], thereby estimating year-specific values of φ and p. As at least one resighting occasion subsequent to a survival period is necessary to calculate separate survival and resighting probabilities [76], parameters pertaining to 1987–2012 were estimable. Bootstrap goodness-of-fit tests showed that this model fitted the data (P = 0.88). The variance inflation factor (ĉ) which quantifies data overdispersion was calculated as the observed model deviance divided by the mean boot strapped deviance (1000 iterations, method a), and as the mean observed ĉ (model deviance divided by the deviance degrees of freedom) divided by the mean boot-strapped ĉ (method b) and by using median ĉ (method c) [78]. For the general CJS model, ĉ was 1.07 (method a) and 1.18 (method b) and median ĉ was 1.14 (method c), indicating minimal overdispersion (values of ĉ = 1.00 indicate perfect fit). A random effects model with random year effects was additionally fitted to φ and p [79–81]. This approach assumes that estimates of φ and p can be modelled as a random variable with variance equal to the process variance. Estimates of φ and p are recalculated given the sample size of individuals contributing to each estimate, and are ‘shrunk’ towards the global mean [80, 81]. Models with random year effects on φ and p were better supported than the fully year-dependent model (Table 1) so results from the random year effects models are presented in figures.

**Table 1:** Candidate CMR models for adult Atlantic Puffin apparent annual survival (φ) and resighting probabilities (p) during 1987 to 2013.

Model no.	Model effect	Model	No par ^b^	QAICc	ΔQAICc ^c^	QDeviance
1	Random year effects on φ	φ(tRYE ^a^)p(t)	43.5	4048.5	0.0	1875.9
2	Random year effects on p	φ(t)p(tRYE ^a^)	52.1	4061.5	13.0	1870.9
3	Split regression of p on years 1987–2013 & 2007–2013	φ(t)p(trends)	29	4062.8	14.3	1920.2
4	Year	φ(t)p(t)	53	4062.9	14.4	1870.5
5	Constant φ	φ(constant)p(t)	28	4064.3	15.8	1923.8
6	Regression of φ on year	φ(trend)p(t)	29	4065.5	17.0	1923.0
7	Regression of φ on Great Skua population size	φ(skua)p(t)	29	4066.3	17.8	1923.8
8	Regression of p on year	φ(t)p(trend)	25	4110.9	62.4	1976.5
9	Regression of p on Great Skua population size	φ(t)p(skua)	26	4208.1	159.6	2071.7
10	Regression of p on breeding success	φ(t)p(breeding success)	26	4239.8	191.3	2103.4
11	Constant p	φ(t)p(constant)	28	4338.8	290.3	2198.3

^a^ Notation ‘RYE’ codes for random year effects models.

^b^ No par = total number of parameters estimated in each model.

^c^ ΔQAICc values are relative to the top ranked model.

The fully year-dependent model was constrained to test hypotheses regarding patterns of temporal variation in φ and p, including in relation to Great Skua population size and Puffin reproductive success. We hypothesised that 1) φ might decrease with increasing Great Skua abundance due to direct predation and hence mortality; 2) p might decrease with increasing Great Skua abundance, via increasing Great Skua abundance reducing the number of Puffins gathering above the colony for normal courtship behaviours due to behavioural avoidance of predation risk, and thereby reducing resighting opportunities; 3) p might increase during years that skua clearance was implemented; and 4) that p might decrease with decreasing Puffin reproductive success because failed breeders might return to sea prior to June and July, precluding resightings. A candidate set of 11 models was generated, which included the fully year-dependent models with and without random effects on φ or p (3 models), constrained models with constant φ or p (2 models), linear regression of φ or p on year (2 models), linear regression of φ and/or p on annual skua population size or annual Puffin breeding success (3 models), and separate linear regression of p over 1987 to 2013 and 2007 to 2013 (1 model).

Akaike’s Information Criterion (AIC) was used to identify the most parsimonious model in the candidate set. AIC values were adjusted to allow for small sample size and for overdispersion measured by ĉ (QAICc). Using AIC to select amongst candidate models obviates problems associated with multiple testing in classical statistics and allows comparison of non-nested models [82,83]. Models that differed by 2 or less QAICc units were considered similarly well supported [82,83]. Puffins are long-lived (up to 30+ years) and individuals ringed in 1986 could potentially have been alive and re-sighted in 2013 [41]. Our dataset therefore provide sufficient power to estimate φ and p despite low ringing effort and relatively low resighting rates in some years (see Results).

### Breeding success

Each year from 1987 to 2013, Puffin breeding success was estimated by marking burrows that contained an egg in May (mean of approximately 84 burrows per year, ranging from 40 in 2004 to 133 in 2010) and re-checking each burrow for the presence or absence of a large chick in July and early August [69,84]. Puffins lay one egg per year and breeding success was measured as the proportion of burrows with an egg that contained a large chick (assumed to fledge) in mid to late July. Breeding success was monitored at study plots on Fair Isle’s north and west cliffs, at Easter Lother, Burrista, South Naaversgill and Greenholm. To minimise potential underestimation or overestimation of breeding success in years when breeding was relatively early, late, or asynchronous, the timing of initial burrow marking was adjusted to coincide with post-laying (judged by checking for eggs at a small sample of burrows (≈ 30) at other accessible parts of the Fair Isle Puffin colony). Up to three subsequent visits were made to assess presence/absence of large chicks through July and early August, with timing adjusted to coincide with large chicks judged from observed rates of adult provisioning.

### Chick diet and provisioning

To quantify among-year variation in Puffin chick food supply, prey loads (1 load = 1 ‘beakful’ of fish) carried by adults feeding chicks were sampled using an 18m mist-net positioned over active nest burrows. Breeding adults returning to their chicks with food flew into the net and dropped their prey loads upon capture (all captured adults were also ringed, measured and released). Sampling was carried out annually from 1987 to 2013 in June to August, in the Toor o’ da Ward Hill sub-colony and with the mist-net positioned consistently. A total of 1008 individual prey loads were collected (annual mean = 37, SD = 21.8), weighed, fish were identified and measured (length in mm) and all prey items identified to one of six prey categories: sandeels *Ammodytidae*, clupeids *Clupeidae*, gadoids *Gadidae*, flatfish *Pleuronectiformes*, pipefish *Syngnathinae* or unidentified/other. Mean annual mass of loads, mass of individual fish in loads, number of fish in loads and the annual proportional representation of prey in the six categories were calculated.

To quantify the rate at which adult Puffins brought prey loads to their chicks, each year from 1989 to 2013, chick provisioning rate in mid-July (number of prey loads delivered per chick per day) was assessed by a full one-day watch of a sample of individually marked burrows each containing a chick (50–100 active burrows at the Roskillie sub-colony). Watches were implemented on or as close as possible to 15^th^ July, by a team of observers watching continuously from 0300 to 2300hrs in calm, dry weather. The number of times adults delivered prey loads to marked burrows was counted, and the total day count then divided by the total number of marked burrows, giving a single rate for the year (number of prey loads delivered per chick per day). This method provides a general annual assessment of chick provisioning rate designed to detect large changes across many years rather than small among-year or within-year variation.

All study plots used for the different components of data collection comprised apparently typical cliff nesting habitat (coastal maritime grassland of short sward height and with deep soil) and were assumed to be representative of the whole island colony and to be similarly exposed to potential predation from Great Skuas and other predators. Breeding Puffins are sensitive to high levels of human disturbance, particularly during incubation [41, 85, 86]. Different plots were used for the different demographic analyses (though for each, the same plots were used consistently for all years), so as to minimise disturbance at any single plot.

### Statistical analyses

Generalized linear models were used to test whether Puffin population size, Great Skua population size, Puffin breeding success and Puffin diet varied among years and to quantify relationships among different measures of diet, and between annual breeding success and mean annual fish load mass. Interpreting the observed association between annual breeding success and mean annual fish load mass is not straight-forward because these two variables both similarly decreased across years (see Results), which could lead to a spurious association. The residuals of regressions of breeding success on year and of mean load mass on year were calculated and correlated (de-trended analysis). However, the probability that the de-trended correlation differed from zero was not estimated because the relatively small sample size of years meant that statistical power of a hierarchical analysis would be low. Models of Puffin and Great Skua population size and of the number of fish in food loads assumed Poisson error distributions. Models of Puffin reproductive performance assumed binomial error distributions. Proportions of different Puffin prey types in food loads were arcsine transformed. Raw means are quoted ± 1 standard deviation (SD) and parameter estimates from models are presented ± 1 standard error (SE). Autocorrelation analyses were carried out on all time-series datasets, but no significant periodicity was detected (P > 0.5 for all lags). Statistical analyses were carried out using R version 2.15.0 (with no additional packages) and generalised linear model, linear model and autocorrelation functions were implemented [87]. CMR models were fitted using program MARK version 6.1 [78].

### Demographic models

**Fig 1:**
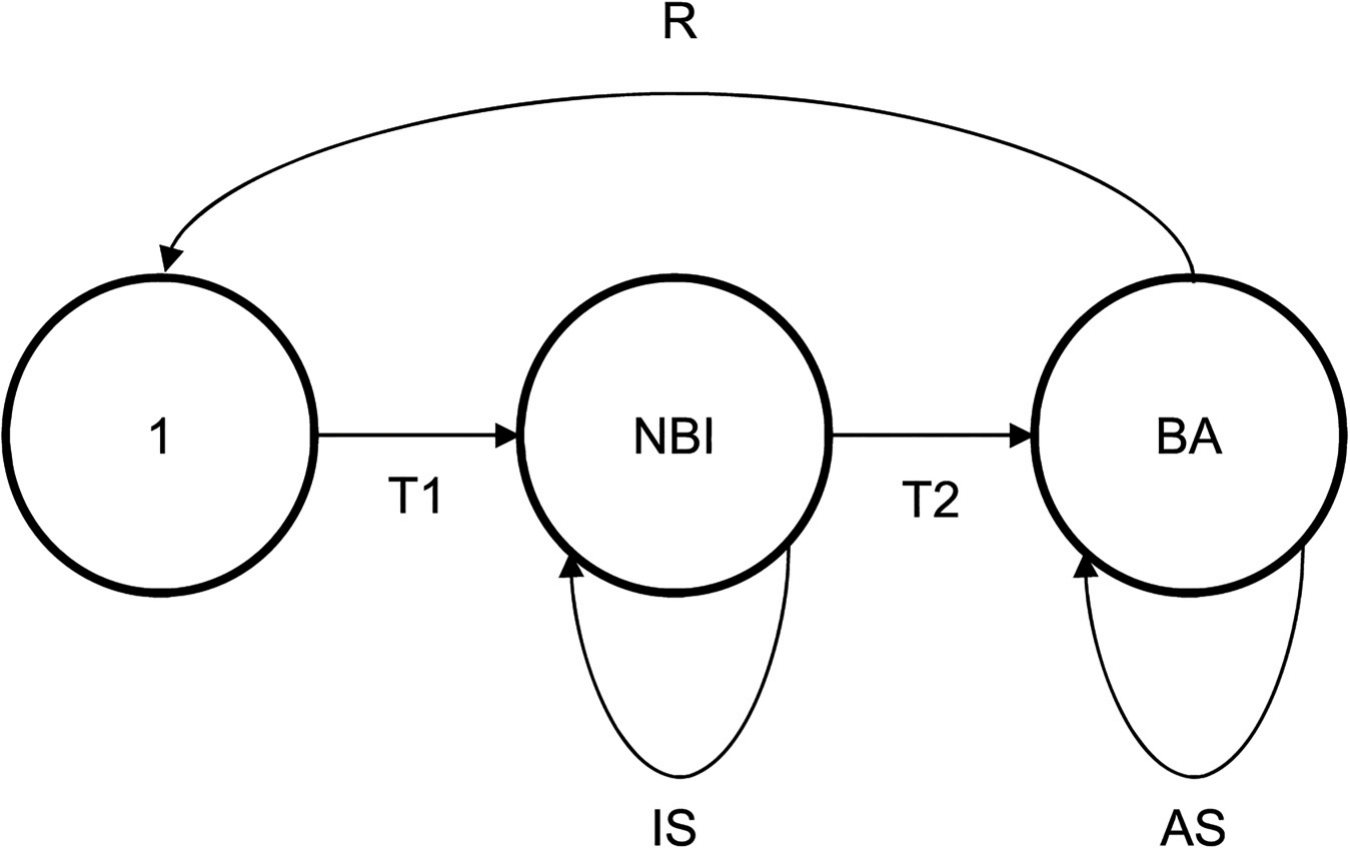
Atlantic Puffin life cycle diagram. Age groupings: (1) one year olds; (NBI) non-breeding immatures, 2 to 5 years of age; (BA) breeding adults. Demographic parameters: (R) breeding success of breeding adult pairs and survival rate of immatures from fledging to one year old [= breeding success of breeding adult pairs x 0.5 x annual survival probability of non-breeding immatures]; (T1) transition rate of immatures from one year old to two year old [= annual survival probability of non-breeding immatures]; (IS) annual survival probability of non-breeding immatures, remaining as non-breeders; (T2) transition rate of immatures to become breeders [= (annual survival probability of non-breeding immatures)^4^]; (AS) annual survival rate of breeding adults, adult Puffin φ.

A simple stage-structured population model founded on the basic life cycle of the Puffin (Fig 1) was used to predict changes in Puffin population size (total number of individuals) during 1987 to 2012 under different demographic conditions, thereby allowing comparisons with changes in real counts across census years. The model included annual adult Puffin φ and the breeding success of breeding adults (i.e. the two demographic parameters estimated on Fair Isle). It also included four other key demographic parameters that were not estimated on Fair Isle: age of first breeding, annual survival probability of non-breeding immatures, annual net rate of immigration and emigration of immatures, and the annual proportion of adults that did not attempt to breed [41, 66, 88]. The model was parameterised for 1987–2012 (i.e. the years for which φ was estimated). The 1987 population size was taken as 21,000 individuals, as an approximate weighted average of the 1986 and 1989 census estimates (see Results). The age of first breeding was fixed as 6 years, matching the mode estimated on the Isle of May, Scotland [41]. Immature survival probability is unknown on Fair Isle but on the Isle of May [89] and at Hornøya, northern Norway [90], did not differ significantly from adult φ. Annual adult Puffin φ estimated on Fair Isle was therefore assumed as a baseline estimate of immature survival probability. All models used estimates of adult Puffin φ for all years from 1987 to 2012 generated from the CMR model with random effects on φ (Table 1). A reference model (Model I) was constructed using the best possible estimates of all parameter values and assuming that immigration and emigration were at equilibrium (i.e. net annual immigration was zero) and that all adult Puffins attempted to breed [69]. Model I was then adjusted to create three new models, in which immature survival probability (Model II), net annual immigration and emigration rate of immatures (Model III) and the proportion of adults that did not attempt to breed (Model IV) were fitted to force the estimates of population size generated by each new model to show a long-term decrease closely matching that shown by the real census counts. Our aim was to determine and evaluate the conditions that would be necessary in each of these three demographic parameters to cause the long-term decrease in population size estimates that was actually observed.

## Results

### Population change

**Fig 2:**
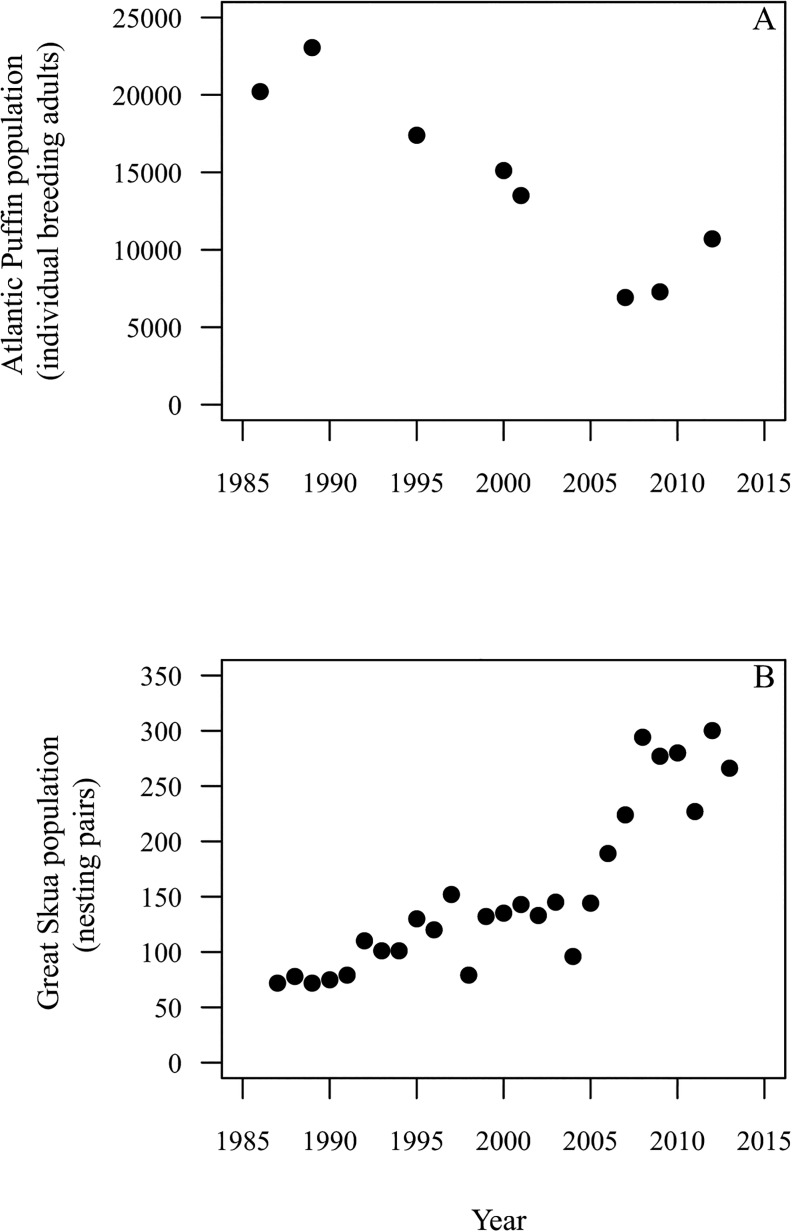
Population size estimates for Atlantic Puffin and Great Skua. A. The maximum count of individual adult Atlantic Puffins on Fair Isle in census years between 1986 and 2013 and B. the total number of Great Skua nesting pairs (apparently occupied territories) on Fair Isle in 1987 to 2013.

During 1986–2012 the maximum number of Puffins counted during day censuses on Fair Isle decreased, approximately halving from an estimated 20,200 individuals in 1986 to 10,700 individuals in 2012. Assessment of Puffin population size on Fair Isle is subject to limitations (see Methods); however, maximum estimates (modelled as absolute values) decreased significantly across years (Fig 2; β = -572.9 ± 0.01 SE individuals per year, z = -117.5, D.F. = 7, P < 0.001).

### Predator abundance and predation of Puffins and auks

During 1987–2013, the number of apparently occupied territories of Great Skuas on Fair Isle increased significantly, from 72 to 266 (Fig 2; β = 8.34 ± 0.02 SE AOT per year, z = 26.7, D.F. = 26, P < 0.001).

In total, 1091 and 814 Great Skua pellets were collected in 2011 and 2012 respectively. Fish was the commonest prey type in both years (comprising 47.9% and 35.4% of prey items respectively; Table 2). Definitively identified Puffins comprised 1.8% in 2011 and 0.5% in 2012. However, unidentified auk species, presumably including Puffins, comprised 27.3% and 23.1% respectively in these years (Table 2).

**Table 2:** Diet composition of Great Skua pairs in 2011 and 2012.

Prey type	Percentage occurrence in Great Skua diet	
	2011	2012
Atlantic Puffin	1.8	0.5
Common Guillemot	0.1	0.1
Razorbill	2.0	0.0
Auks (species unidentifiable)	27.3	23.1
Black-legged Kittiwake	5.7	1.4
Northern Fulmar	2.0	0.9
European Storm Petrel	0.0	0.1
European Shag	0.3	0.1
Great Skua chick	2.3	0.1
Seabird egg	0.2	21.7
Unidentified / other bird	0.3	5.5
Fish	47.9	35.4
Mammal	8.2	10.1
Other	1.8	1.0

### Adult survival and resighting probability

**Fig 3:**
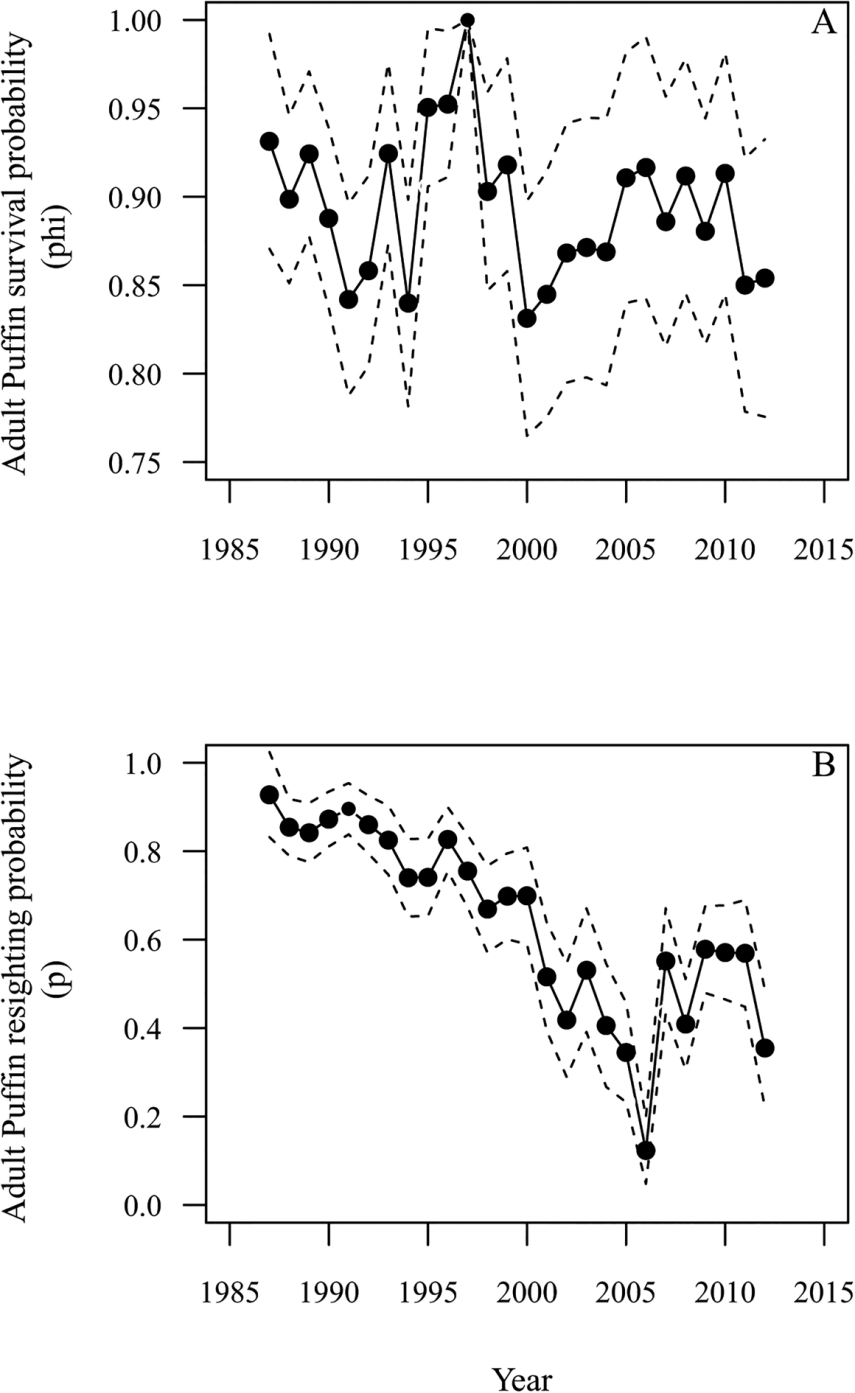
Atlantic Puffin survival and resighting probability estimates. A. Mean estimated adult Atlantic Puffin apparent survival probability (φ) and B. resighting probability (p) during 1987 to 2012. Estimates of φ and p are from models with random year effects on φ and p (Table 1 and see Methods). Dashed lines represent ± 95% confidence intervals.

The best supported CMR model was the fully year-dependent model with random year effects on φ (Table 1, Fig 3). The model with random year effects on p, the fully year-dependent model in φ and p and the model with φ constrained to be constant were similarly well supported but less well supported than the best model (Table 1, differences in ΔQAICc values < 2). Models with linear regression of φ on years, Great Skua population size or Puffin reproductive success were less well supported than the fully year-dependent model (Table 1, differences in ΔQAICc values > 2).

Models with constant p, or with a linear regression of p on year, annual Great Skua population size or Puffin reproductive success were all less well supported than the fully year-dependent model and the model with random year effects on p which were similarly well supported (Table 1, differences in ΔQAICc values < 2). Estimates of p from the fully year-dependent model with random year effects decreased substantially during 1987 to 2006, from 0.93 in 1987 to 0.12 in 2006, increased in 2007 and then ranged between 0.58 and 0.36 subsequently (Fig 3). The model with a linear regression of p on year was better supported than the model with constant p, further supporting the strong decrease in p estimated by the fully year-dependent model (Table 1).

### Behaviour, resighting probability and Great Skua clearance

The constrained model with separate linear regressions of p on years 1987 to 2013 and 2007 to 2013 was as well supported as the fully year-dependent model (differences in ΔQAICc < 2) and better supported than all other candidate models, except for the fully year-dependent models with random year effects on φ and p (Table 1). In years in which Great Skua clearance was implemented (2007–12), mean p was 0.51 ± 0.11 SD, compared with 0.29 ± 0.15 SD in 2004–06. However, this did not represent a complete recovery to the values estimated during 1987–1996 (mean p = 0.84 ± 0.06 SD; Fig 3).

### Breeding success

**Fig 4:**
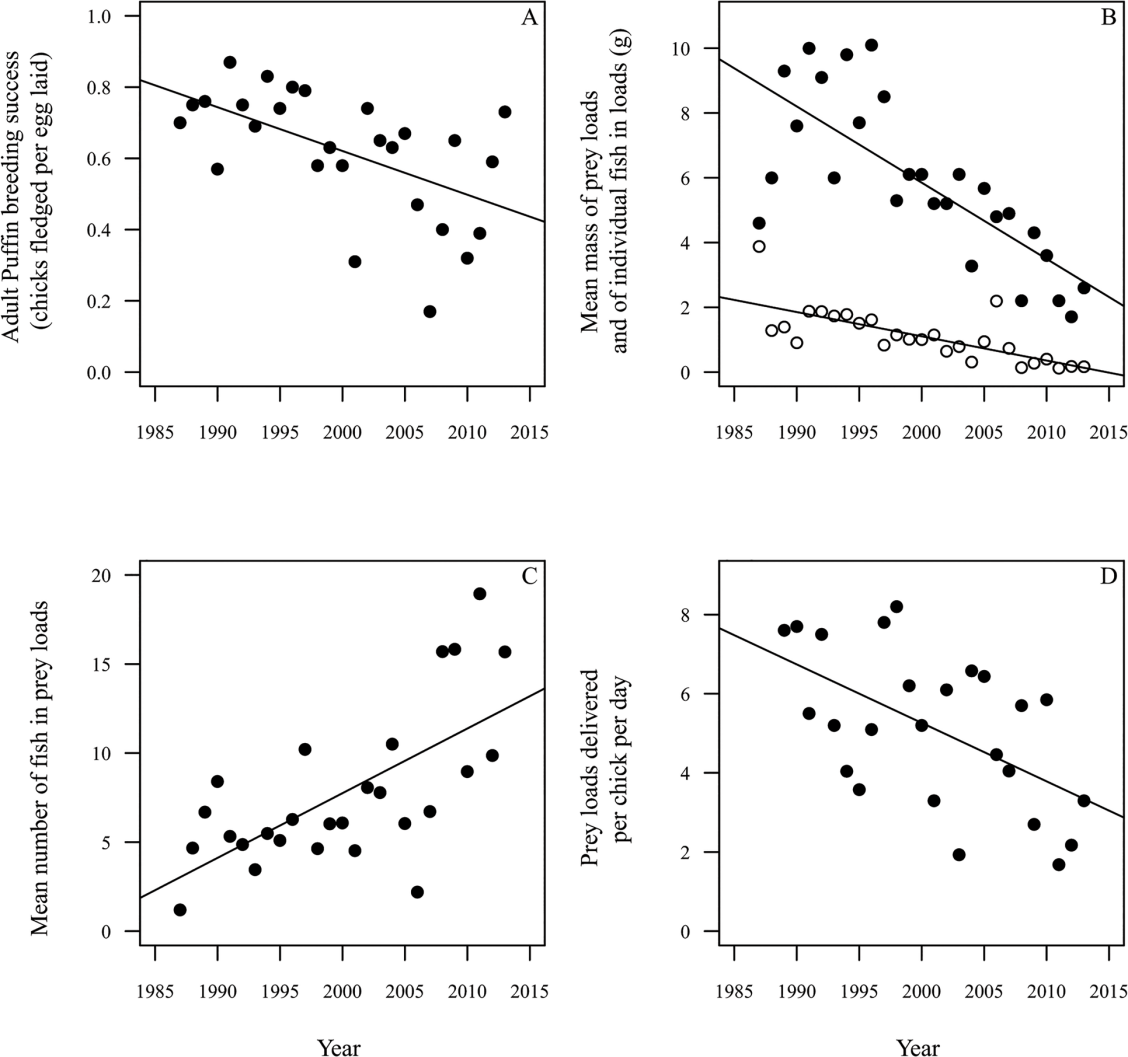
Atlantic Puffin breeding success and chick diet characteristics. A. Adult Atlantic Puffin breeding success, 1987 to 2013; B. mean total mass of prey loads landed by adult Atlantic Puffins for chicks, filled circles, and mean mass of individual fish in loads, open circles, 1987 to 2013; C. mean number of fish in loads, 1987 to 2013; D. number of fish loads delivered per chick per day in mid-July, 1989 to 2013. Lines represent fitted linear regressions.

Mean Puffin breeding success during 1987 to 2013 was 0.62 ± 0.18 SD chicks fledged per egg laid. Breeding success decreased significantly across years (Fig 4; t = -3.8, D.F. = 26, P < 0.001), with an annual decrease of β = -0.01 ± 0.02 SE chicks fledged per egg laid per year.

### Chick diet characteristics

The mean mass of prey loads landed by adult Puffins for chicks between 1987 and 2013 was 5.9g ± 2.5 SD. Load mass decreased significantly across years (Fig 4; t = -5.7, D.F. = 25, P < 0.001), by β = -0.24 ± 0.04 SE grams per year, in total equating to a 68% decrease in mean annual load mass across the study period. The mean mass of individual fish in these loads (fish mass) was 1.1 ± 0.8 SD grams and decreased significantly across years (Fig 4; t = -5.2, D.F. = 25, P < 0.001) by β = -0.07 ± 0.01 SE grams per year. The mean number of fish in these loads (fish number) increased significantly across years (Fig 4; z = 5.21, D.F. = 26, P < 0.001) by β = 0.36 ± 0.01 SE fish per year. The mean number of prey loads delivered per chick per day in mid-July by adult Puffins (feeding frequency) during 1989 to 2013 was 5.1 ± 1.9 SD but decreased significantly across years (Fig 4; t = -3.2, D.F. = 23, P = 0.004) by β = -0.15 ± 0.05 SE loads delivered per chick per day per year, in total equating to a 51% decrease in feeding frequency across the study period.

Sandeels, gadoids and clupeids were observed in Puffin prey loads in 26, 25 and 18 of the 26 study years respectively (Table 3). Pipefish occurred only between 2004 and 2007 (maximum representation = 41.2% in 2006) and flatfish were encountered very rarely, with small proportional representation (< 5%) in only 1988, 2001, 2002 and 2006. Proportional representation of the six prey categories in prey loads varied among years (Table 3) and on average sandeels accounted for the greatest percentage (27 year mean representation = 57.1%, SD = 25.9) then gadoids (30.7%, SD = 22.8) and clupeids (8.7%, SD = 16.2). None of the six prey categories significantly increased or decreased in proportional representation across years (sandeels, β = -0.78 ± 0.52 SE % per year, t = -1.6, D.F. = 25, P = 0.13; gadoids, β = 0.27 ± 0.36 SE % per year, t = 0.4, D.F. = 25, P = 0.69; clupeids, β = 0.31 ± 0.25 SE % per year, t = 0.8, D.F. = 25, P = 0.45; flatfish, β = 0.01 ± 0.01 SE % per year, t = 0.3, D.F. = 25, P = 0.74; pipefish, β = 0.19 ± 0.12 SE % per year, t = 1.0, D.F. = 25, P = 0.34; unidentified prey, β = -0.01 ± 0.06 SE % per year, t = -0.1, D.F. = 25, P = 0.98).

**Table 3:** The number of prey loads landed by adult Atlantic Puffins, number of individual fish in loads and the proportional occurrence of different prey types in loads during 1987 to 2013.

Year	Total fish loads collected	Total individual fish in all loads	Percentage representation among total individual fish in all loads					
			Sandeels	Gadoids	Clupeids	Flatfish	Pipefish	Unidentified / other
1987	27	32	100	0	0	0	0	0
1988	34	159	42.3	52.1	5	0.6	0	0
1989	64	428	74.3	16.5	9.2	0	0	0
1990	73	613	45.2	41.6	13.2	0	0	0
1991	72	384	93.2	3.9	2.9	0	0	0
1992	107	520	28.8	71.2	0	0	0	0
1993	15	52	44.3	51.9	3.8	0	0	0
1994	18	99	82.8	16.2	0	0	0	1
1995	28	143	55.9	36.4	7.7	0	0	0
1996	26	163	88.4	6.1	5.5	0	0	0
1997	44	449	40.6	54.4	1.3	0	0	3.7
1998	57	264	17.8	77.3	0	0	0	4.9
1999	32	193	95.9	3.1	1	0	0	0
2000	38	231	97	0.9	2.1	0	0	0
2001	19	86	63.2	13.7	2.3	1.2	0	19.6
2002	30	242	40.5	12.8	43.4	3.3	0	0
2003	33	257	14.8	18.7	66.5	0	0	0
2004	6	63	84.1	14.3	0	0	1.6	0
2005	25	151	53.9	7.3	36.2	0	2.6	0
2006	31	68	7.4	39.6	10.3	1.5	41.2	0
2007	42	282	60	32.6	0	0	7.4	0
2008	36	565	48.5	51.5	0	0	0	0
2009	6	95	34.7	65.3	0	0	0	0
2010	35	314	62.9	36.2	0.9	0	0	0
2011	33	625	63.7	36.3	0	0	0	0
2012	37	365	52.5	47.2	0.3	0	0	0
2013	40	627	48.9	21.6	29.3	0	0	0.2
Mean ^**a**^	37.3	276.7	57.1	30.7	8.9	0.2	1.9	1.1
S.D.	21.8	190.6	25.9	22.8	16.2	0.7	7.9	3.9

^**a**^ Mean values were derived by giving equal weight to all years

### Breeding success and chick diet

**Fig 5:**
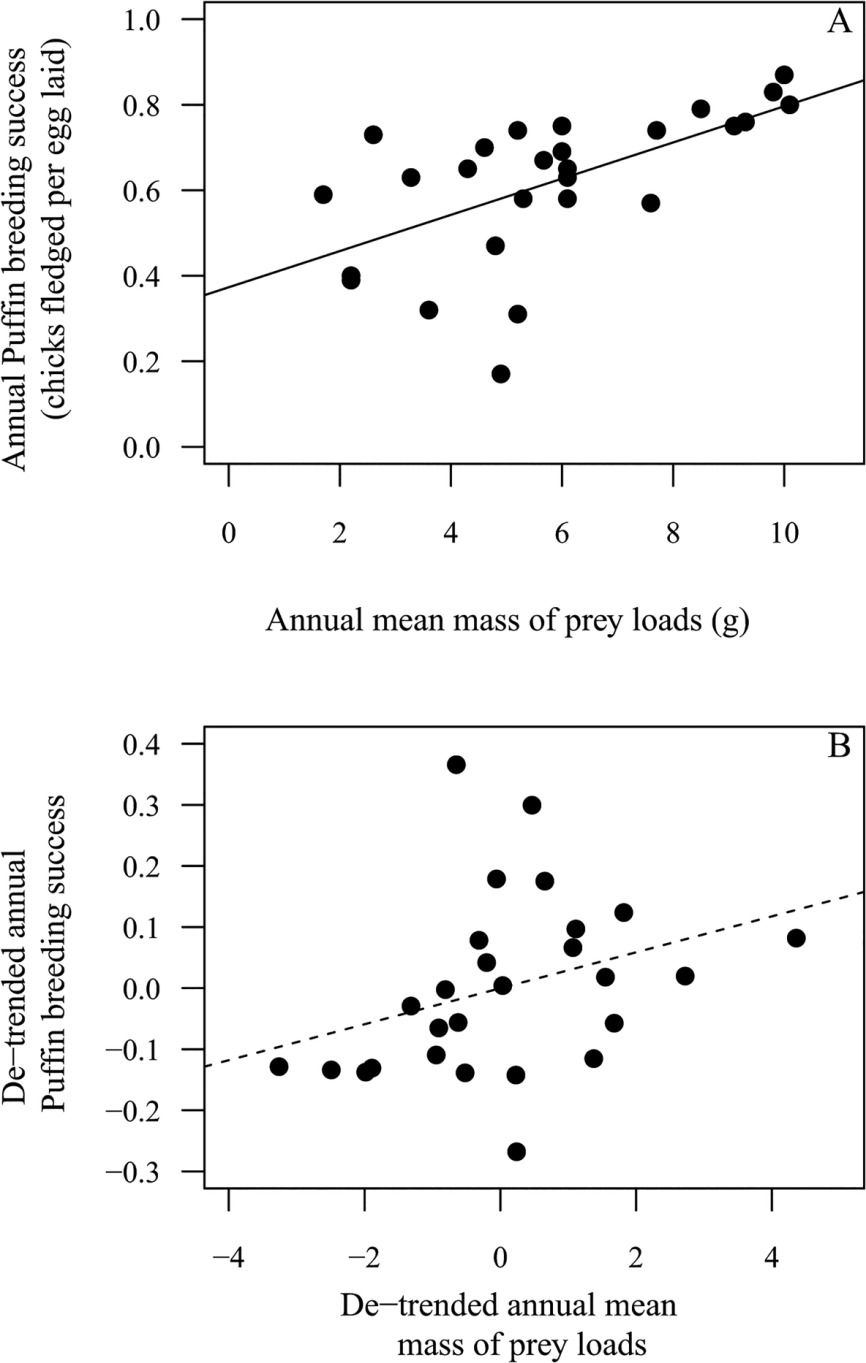
Relationships between Atlantic Puffin breeding success and annual mean mass of fish loads delivered to chicks. A. Values of annual adult Atlantic Puffin breeding success and annual mean mass of fish loads delivered to chicks in the same year during 1987 to 2013 plotted against each other without de-trending and B. plot of de-trended residuals. Lines represent fitted linear regressions (solid line = significant positive correlation, stippled line = weak positive correlation, non-significant).

Puffin breeding success was positively correlated with annual mean prey load mass (z = 11.4, D.F. = 26, P < 0.001), with an increase of β = 0.04 ± 0.02 SE chicks fledged per gram in annual mean mass of fish loads (Fig 5). The residuals from the regression of breeding success on year were only weakly positively correlated with the residuals from the regression of mean load mass on year (de-trended analysis: r = 0.09; Fig 5).

### Demographic modelling

The reference model that used all best possible parameter estimates (Model I), predicted rapid population growth (λ = 0.07, Table 4) and therefore did not match the substantial decrease in census counts (λ = -0.03). In order to reproduce the change estimated by census data, annual immature survival rate would have to be 0.74 (Model II; Table 4), or net annual immigration and emigration rate of immatures would have to be -68% (Model III), or the annual proportion of adults that did not attempt to breed (Model IV) would have to increase dramatically across years, from 13.4% in 1988 to 86.4% in 2012 (Table 5).

**Table 4:** Definitions of Atlantic Puffin demographic model terms and specified parameter values.

		Model			
Parameter	Definition	I	II	III	IV
φ 1	Adult survival probability	Annual estimates from Fair Isle from CMR1 ^a^	Annual estimates from Fair Isle from CMR1 ^a^	Annual estimates from Fair Isle from CMR1 ^a^	Annual estimates from Fair Isle from CMR1 ^a^
m	Breeding success of an adult that attempted to breed	Annual estimates from Fair Isle	Annual estimates from Fair Isle	Annual estimates from Fair Isle	Annual estimates from Fair Isle
φ 2	Immature survival probability (age 1 to age 5)	Annual estimates of adult survival probability from Fair Isle from CMR1 ^a^	0.74 (constant, fitted value)	Annual estimates of adult survival probability from Fair Isle from CMR1 ^a^	Annual estimates of adult survival probability from Fair Isle from CMR1 ^a^
e	Net immature emigration and immigration (% change)	0	0	-68 (constant, fitted value)	0
c	Proportion of adults that did not attempt to breed	0 (constant, fitted value)	0 (constant, fitted value)	0 (constant, fitted value)	(fitted values given in Table 5)
λ	Proportional change in population size per year	+0.07	-0.03 ^b^	-0.03 ^b^	-0.03 ^b^

^a^ CMR1 = capture-mark-recapture model 1, see Table 1.

^b^ A linear model fitted to population size estimates from real census counts (1986 to 2013) gave proportional change in population size per year (λ) as -0.03.

**Table 5:** The estimated proportion of adult Atlantic Puffins that would not attempt to breed each year, if the estimated population decrease on Fair Isle (λ = -0.03) were to be caused entirely by permanent or intermittent non-breeding and consequent failure to observe surviving individuals.

Year	Adults not breeding (%)	Year	Adults not breeding (%)
1988	13.4	2001	70.4
1989	22.9	2002	72.7
1990	30.0	2003	74.8
1991	35.5	2004	76.3
1992	39.9	2005	77.9
1993	43.5	2006	79.3
1994	47.6	2007	80.1
1995	51.8	2008	82.0
1996	54.8	2009	83.4
1997	59.1	2010	84.7
1998	62.3	2011	85.9
1999	65.0	2012	86.4
2000	68.1		

## Discussion

This study aimed to evaluate the magnitude and mechanisms of changes which occurred in a Puffin population studied since 1986 as part of the UK national seabird monitoring program. The estimated number of breeding adult Puffins decreased markedly, by approximately 50% during 1986 to 2012. Similar decreases in this species have been observed at other northeast Atlantic colonies (e.g. Shetland and Faeroes) and in this region populations of many other seabird species have also decreased since 1986 [26, 27]. The estimated population decrease observed during this study must result from some combination of demographic rates, ecological factors and, potentially, observation failure during censuses. We next evaluate these possibilities.

### Survival and resighting probabilities

Estimated adult Puffin survival probability (φ) averaged 0.89 ± 0.05 SD, and did not decrease or increase across study years. This estimate is slightly lower than at other colonies in the UK, Norway and North America, where φ averaged between 0.90 and 0.95 [91, 92]. This could reflect localised variation in food availability or predator abundance.

The breeding population of Great Skuas increased substantially during 1987 to 2013, approximately trebling, and increasing from 120 AOTs in 1996 to 300 in 2012. Diet analyses in 1996, 2003, 2011 and 2012 demonstrated considerable predation of seabirds. Although pellets classified as ‘auks’ likely included Puffins, the exact representation is unknown. Overall, predation pressure on seabirds is likely to have increased considerably during the study period. However, φ for adult Puffins did not show the predicted decrease. There was therefore no clear evidence of a detrimental top-down effect of increasing Great Skua numbers on adult Puffin survival.

Puffin movements from Fair Isle have not been tracked, meaning that summer foraging and wintering locations are unknown. Without this information, it is not possible to assess how adult survival may be influenced by bottom-up ecological drivers such as fish and plankton stock characteristics and environmental conditions in relevant locations. Other studies have shown that oceanic and other predators can experience bottom-up control of survival rates by effects of changing environmental conditions and food availability [93–96]. However, since φ remained relatively high and stable throughout the study period there was no evidence of a reduction as could be caused by deteriorating food availability.

Estimated resighting probability (p) decreased markedly during 1987 to 2012. This is unlikely to reflect changes in observer effort as re-sighting regimes remained broadly consistent across years. Rather, it probably reflects increasing observation failure caused by the reduced time for which Puffins were visible on land. Specifically, the frequency which adults returned to provision chicks decreased from an approximate average of 7 to 3 fish load deliveries per chick per day. Furthermore, having gathered on the colony during the 1980s and 1990s, adult Puffins subsequently switched to gathering mostly on the sea.

We hypothesised that the decrease in p for adult Puffins was associated with the increase in Great Skua numbers from 1986 to 2013. The CMR model with p constrained to vary with annual skua population size was less well supported than the fully year-dependent model. However, Great Skua clearance implemented since 2007 was associated with an increase in p. This in turn implies that gathering on the sea was a behavioural plasticity by Puffins to avoid skuas on the colony. This would not have influenced population size estimates via observation failure because in years when Puffins gathered on the sea rather than on land, invariably they were very close inshore below the colony, less than 200m from the cliff base, and the census method included counting all individuals within this distance. Predation-avoidance against large skua species has been described from the southern hemisphere, where skuas very heavily depredate small seabirds [97–100]. Equivalent observations in the northern hemisphere are very rare, possibly because the only large skua is the Great Skua, which has a relatively limited distribution and until recently has fed extensively on fish, including fisheries discards [46, 68, 101].

Great Skua clearance during 2007 to 2012 did not, however, result in p values as high as in early study years, implying that the observed decrease in p between 1987 and 2006 was not solely due to skua-induced changes in Puffin gathering behaviour. This discrepancy likely reflects the decrease in chick provisioning rate, and hence reduced frequency of colony visits, by approximately 60% between 1989 and 2013. It might also imply that a proportion of adult Puffins skip breeding seasons [102, 103]. However, such intermittent non-breeding by experienced adult Puffins has previously been estimated to happen in only a very small minority of individuals [41]. Over a nine-year period on the Isle of May there were only 27 cases of definite non-breeding among over 2,000 bird-years [41]. Chick feeding frequency remained high and stable on the Isle of May during 1985 to 2013 and resighting probability was consistently high during this period, with no decrease or increase (M. Harris pers. comm.) [41].

### Breeding success and chick prey characteristics

Puffin breeding success decreased substantially on Fair Isle during 1987 to 2013. Temporal variation in vertebrate breeding success has been widely linked to variation in the availability and characteristics of food provisioned to young [14, 104–108]. The mass of prey loads delivered to Puffin chicks decreased considerably from 1987 to 2013, as did individual fish mass and chick feeding frequency. Never was any widespread evidence found of kleptoparasitism of adult Puffins by Great Skuas and gulls, Puffin nest burrows being dug out by Great Skuas or other predators, predation of unfledged chicks by Great Skuas or other predators, or of disease or parasite infestations among Puffin chicks, so it seems unlikely that on Fair Isle declines in breeding success were driven more by these factors than by bottom-up ecological effects, namely decreases in prey load mass, fish mass and feeding frequency.

A possible mechanism by which the observed changes in chick diet characteristics could be explained is that between 1987 and 2013 the size of all available prey fish decreased where adult Puffins forage. Therefore smaller and smaller fish were caught and higher total numbers, but this takes more time at sea so chick feeding frequency decreased. At the Isle of May the length of 0-group sandeels brought ashore by Puffins was highly correlated with the length of 0-group sandeels caught on the same day by research vessels sampling fish stocks within the bird’s foraging range [109]. Observed changes in chick diet characteristics in our study may similarly reflect changes in fish stock characteristics at sea.

Fish mass is not always proportionally representative of the nutritional value of the fish [33]. Indeed, energy value of Lesser Sandeels and Sprats *Sprattus sprattus* brought ashore for chicks by Common Guillemots at the Isle of May was sometimes unexpectedly low, and independent of fish size. This exacerbated negative effects of low prey mass and was a probable cause of breeding failure [33]. Were fish to be of relatively high energy value at Fair Isle this is unlikely to compensate for the substantial observed decreases in the mass and delivery rate of prey loads. To fully elucidate the situation however, measurement of energy values and representation in the diet (by mass) of different prey types is necessary. In this study the observed decrease in mean annual mass of prey loads delivered to chicks could not be attributed to changes in mass of any specific prey types, for example sandeels, because the mass of individual fish was not measured and individual prey loads usually comprised more than one prey type. The proportional occurrence (by number) of sandeels, gadoids or clupeids brought ashore by adults did not change significantly across the study years, in most years all three were recorded as prey items, and from this there was no evidence that changes in prey characteristics may be attributable to changes in one prey type.

It remains uncertain whether breeding failures on Fair Isle occurred due to chick mortality or failure at egg stage. The methodology used to measure breeding success aimed to minimise disturbance and involved one colony visit just after eggs were laid and then further visits once the chicks were relatively large, so the survival or failure of eggs and chicks was not closely tracked. It is impossible to estimate at what point in the season failed breeders might have left the colony, but the CMR model in which resighting probability was constrained to vary with breeding success was less well supported than the fully year-dependent model, implying that any effects on resighting probability of failed breeders that had returned to sea were small.

Our results imply that adult Puffins maintain their own survival at the expense of breeding success. In a relatively long-lived species with low fecundity this might be expected, because even a small reduction in adult survival would reduce the number of subsequent breeding attempts and hence reduce lifetime breeding success [110, 111].

### Mechanisms of population decrease

In long-lived vertebrates, in particularly those with delayed sexual maturity and low fecundity, adult survival has the potential to be the main factor influencing population growth rate [8, 10, 112–114]. Basic parameterisation of a demographic model using parameter values estimated on Fair Isle or the best available estimates from other studies predicted positive population growth. This prediction contrasts with the decrease in census counts and implies that one or more parameters or population size was poorly estimated. Adult survival probability and reproductive success were adequately estimated, implying that these parameters were not the primary cause of the observed population decrease.

We modelled three scenarios that would account for a population decrease matching that indicated by census counts: that immature survival probability was low (approximately 0.74 rather than equal to annual adult survival probability); that a high proportion of immature birds permanently emigrate every year (approximately 68%, rather than net immigration and emigration rate being zero); and that an increasingly high proportion of the adult population temporarily does not breed and is therefore absent from Fair Isle during censuses.

In Puffins, immature survival is very high at the Gulf of Maine, Isle of May and Hornøya, where it was 0.85, 0.93 and 0.93 respectively [89–91, 115]. In comparison, a value of 0.74 represents a substantial reduction from the mean adult survival probability of 0.89 estimated on Fair Isle. However, age-related differences in survival are not impossible, especially considering that the foraging and wintering locations of Puffins from Fair Isle are unknown but may differ between adults and immatures and that immature Puffins could be more susceptible to predation than adults, as has been found in other marine birds [46, 68, 99, 100, 116, 117].

Rates of Puffin emigration and immigration to and from different colonies are largely unknown. In 1969–70, 20,000 adult and young Puffins were marked at 15 sites in the UK using colony-, age- and breeding state-specific ring combinations [41]. Most resighted birds were only recorded at the colony where they were ringed, although some birds visited nearby colonies and movement rates were variable. On the Isle of May for example, 62% of surviving young emigrated [41]. Similarly, in a study at the Gulf of Maine, up to 57% of young Puffins emigrated from natal colonies [37]. High immature emigration, as suggested by Model III, therefore could potentially explain the population decrease on Fair Isle.

Modelling of non-breeding among adults as the cause of the estimated population decrease required the proportion of non-breeding adults to be >20% from 1988, >50% from 1995 and > 80% from 2007 (Table 5). Such substantial rates do not seem plausible given that breeding Puffins are highly site-faithful, usually returning to the same burrow every year [41], and there was no evidence to suggest temporary desertion of burrows on Fair Isle. Furthermore, both the decrease in chick provisioning rate by approximately 60% between 1989 and 2013 and the comparatively high resighting probabilities in years when Great Skuas were cleared imply that rather than the majority of Puffins being entirely absent during the latter years of the study, most were present but just spending less time on the colony.

Finally, one other potential driver of population change is change in modal age of first breeding. However, even if the modal age of first breeding had increased by 1 year of age annually between 1987 and 2013 this would still not generate a decrease in breeding population size to match the real census data.

Clearly Great Skuas exert predation pressure on seabirds, and if immatures rather than adults were predated this could explain why adult Puffin survival probability remained stable from 1987 to 2012. Predation of naïve, young prey rather than older, experienced individuals has been observed extensively in marine and terrestrial systems, but in seabirds is generally very difficult to conclusively determine due to the similarity of adults and fully grown young [46, 68, 118, 119]. On the Isle of May, the presence of large, predatory, breeding gulls had a significant negative effect on the recruitment of Puffins to the colony [120], and the presence of Great Skuas on Fair Isle could possibly have a similar effect. Additionally, changes in Puffin chick diet characteristics between 1987 and 2013 suggest that prey quality for Puffins on Fair Isle has decreased, which could conceivably cause emigration by immature birds to sites where conditions are better.

Low immature survival and high immature emigration rates are the most plausible scenarios to explain the population decrease on Fair Isle. From this study, it is not possible to determine the relative influence of each of these parameters but ultimately both limit recruitment. We conclude that a decrease of approximately 50% occurred in the Puffin population on Fair Isle between 1986 and 2012 and this was probably due to a combination of factors, but that depressed recruitment of immatures into the breeding population was the most likely primary mechanism of change.

This study provides an example of holistic evaluation of the evidence for, and mechanisms causing, a major decline in a seabird population in a region where many seabird populations have recently declined. It shows that increase in the size of a predator population does not always impact on the survival of adult prey, that a decrease in breeding success does not always cause decrease in colony size, and that consideration of behavioural changes, potentially driven by changing ecological factors such as predation pressure and food supply, is crucial in assessment of estimates of population size. For conservation managers, these are critical considerations, without which predator effects and strategies to mitigate population declines could easily be misjudged.

## Supporting Information

S1 Table Atlantic Puffins colour-ringed and resighted. The number of breeding adult Atlantic Puffins marked using colour-rings and resighted in years from 1986 to 2013.
(DOCX)
