# Supplementary material for: Decline in an Atlantic Puffin Population: Evaluation of Magnitude and Mechanisms
Source: PLoS One. 2015 Jul 15;10(7):e0131527. doi: 10.1371/journal.pone.0131527 (PMC4503501; doi:10.1371/journal.pone.0131527)
Supplement: S1 Table — The number of breeding adult Atlantic Puffins marked using colour-rings and resighted in years from 1986 to 2013. (DOCX) [file pone.0131527.s001.docx]

**S1 Table. Atlantic Puffins colour-ringed and resighted.** The number of breeding adult Atlantic Puffins marked using colour-rings and resighted in years from 1986 to 2013.

|  |  |  |  |  |  |  |  |  |
| --- | --- | --- | --- | --- | --- | --- | --- | --- |
| Year | Marked ^a^ | Resighted ^b^ | Year | Marked ^a^ | Resighted ^b^ | Year | Marked ^a^ | Resighted ^b^ |
|  |  |  |  |  |  |  |  |  |
| 1986 | 33 | 0 | 1996 | 1 | 89 | 2006 | 6 | 11 |
| 1987 | 111 | 29 | 1997 | 4 | 82 | 2007 | 36 | 44 |
| 1988 | 14 | 109 | 1998 | 26 | 68 | 2008 | 31 | 44 |
| 1989 | 22 | 111 | 1999 | 0 | 84 | 2009 | 14 | 69 |
| 1990 | 18 | 119 | 2000 | 15 | 66 | 2010 | 33 | 72 |
| 1991 | 13 | 114 | 2001 | 0 | 46 | 2011 | 11 | 73 |
| 1992 | 3 | 102 | 2002 | 7 | 32 | 2012 | 8 | 38 |
| 1993 | 11 | 94 | 2003 | 0 | 37 | 2013 | 1 | 50 |
| 1994 | 4 | 75 | 2004 | 39 | 23 |  |  |  |
| 1995 | 11 | 75 | 2005 | 5 | 30 |  |  |  |
|  |  |  |  |  |  |  |  |  |
|  |  |  |  |  |  |  |  |  |

^a^ The number of individuals marked using unique combinations of 3 colour and 1 metal rings.

^b^ The number of individuals resighted that had been marked in previous years.
